# Prevalence and incidence of non-communicable diseases among older adults living with HIV on antiretroviral therapy in Northern Haiti: A retrospective cohort study

**DOI:** 10.1371/journal.pgph.0007175

**Published:** 2026-10-01

**Authors:** Ronald Thiersaint, Romaine Nephtalie Mésidor, Valérie Pelletier, Chesnel Parny Norcéide, Jean Géto Dubé, Harold Prévil, Kesner François, Jean Baptiste T. Koama, Michael A. Melchior, Macarthur Charles

**Affiliations:** 1 U.S. Centers for Disease Control and Prevention, Port-au-Prince, Haiti; 2 Catholic Medical Mission Board, Port-au-Prince, Haiti; 3 Unité de Gestion de Projets, Ministère de la Santé Publique et de la Population, Port-au-Prince, Haiti; 4 Hôpital Universitaire Justinien, Cap Haïtien, Haiti; 5 Hôpital Sacré-Cœur de Milot, Cap Haïtien, Haiti; 6 Programme National de Lutte contre les IST/VIH/SIDA, Ministère de la Santé Publique et de la Population, Port-au-Prince, Haiti; 7 U.S. Centers for Disease Control and Prevention, Atlanta, Georgia, United States of America; University of the Witwatersrand Johannesburg Faculty of Health Sciences, SOUTH AFRICA

## Abstract

The growing burden of non-communicable diseases (NCDs) among aging people living with HIV (PLHIV) represents a major public health challenge, yet data to guide integrated care in Haiti remain limited. To address this gap, we conducted a retrospective cohort study of PLHIV aged 40 years and older receiving care at two hospitals in northern Haiti. Using electronic medical record data from routine HIV clinical visits between July 1 and September 30, 2015, we compared NCD prevalence at ART initiation and after ≥12 months of follow-up. We also identified baseline risk factors associated with incident NCDs, with a secondary analysis focused specifically on predictors of incident renal insufficiency. Among 318 participants, 65.7% had one or more NCD at ART initiation, increasing to 70.8% after ≥12 months. The prevalence of hypertension rose from 37.1% to 47.2% and diabetes from 7.9% to 12.6%, while overweight/obesity remained stable (14.5% to 15.7%). In contrast, renal insufficiency prevalence decreased from 31.1% to 22.6%. In the adjusted analysis, female sex was the sole independent predictor for developing at least one incident NCD (AOR: 3.29; 95% CI: 1.40–7.72). In the secondary analysis for incident renal insufficiency, female sex (AOR: 5.88; 95% CI: 1.78–19.50) and elevated systolic blood pressure (AOR: 1.052; 95% CI: 1.005–1.101) were the only significant predictors. A high proportion of older PLHIV in northern Haiti are affected by NCDs. These findings underscore the need for integrated care models, particularly for older women living with HIV, who appear to be at the highest risk for developing new NCDs.

## Introduction

In 2003 the Haiti Ministry of Health (MOH) undertook one of the most important steps in the fight against HIV by ensuring the availability of antiretroviral therapy (ART) for people living with HIV (PLHIV). As of 2024, with support from the U.S. President’s Emergency Plan for AIDS Relief (PEPFAR) and from The Global Fund to Fight AIDS, Tuberculosis, and Malaria, ~ 146,000 PLHIV are receiving ART free of charge around the country [1]. The scaling up of ART and associated HIV prevention services in Haiti have significantly reduced morbidity and mortality associated with HIV and AIDS [2,3].

In 2016, the MOH adopted the “Test and Treat” strategy – offering immediate ART to all individuals who test positive for HIV – to further reduce HIV-related morbidity and mortality and achieve epidemic control [4]. As more PLHIV are linked to care and start treatment earlier, and as those on ART grow older, an increasing number of PLHIV will confront age-related non-communicable diseases (NCDs), including cardiovascular disease, kidney and liver disorders, and cancers. In 2024, PLHIV aged 40 years and older accounted for 54% of all PLHIV on ART across PEPFAR-supported countries globally, while in Haiti, this age group represented 58% of PLHIV actively receiving ART [5].

Prolonged exposure to ART along with aging and HIV infection itself may increase the risk of developing metabolic complications and cardiovascular disease among PLHIV [6,7]. NCDs such as diabetes mellitus, hypertension, coronary artery disease, hyperlipidemia, renal disease, and reduced bone mineral density are more prevalent among elderly PLHIV-compared to their HIV-uninfected counterparts [8,9]. Managing ART can be challenging if the person living with HIV is also experiencing other chronic conditions. While older PLHIV generally demonstrate higher ART adherence than younger individuals [10,11], managing concurrent chronic conditions presents significant challenges. Patient adherence often declines not only due to the complexities of polypharmacy, but also because of structural health system constraints. In Haiti, HIV care models feature varying levels of NCD integration; while routine NCD screening is generally incorporated into HIV clinic visits, subsequent care ranges from direct on-site management to external referrals, depending on the specific NCD and facility capacity. Navigating these varied and sometimes fragmented care pathways can place an additional operational burden on aging patients, further complicating their long-term management.

At an estimated 427.7 deaths per 100,000 population, Haiti has the highest age-adjusted cardiovascular disease mortality rates in the Americas Region [12]. However, despite this high overall population-level burden, there is a critical lack of NCD data disaggregated by HIV status, age, and region. Consequently, the specific burden of NCDs among PLHIV aged 40 years and older in Haiti remains largely understudied. This is particularly true for the North department, where no studies on NCDs have previously been performed, despite the region being home to approximately 13% of the 140,000 PLHIV on ART in Haiti [13]. Although guidelines have recently been updated to recommend annual screening and monitoring for NCDs in PLHIV, conditions like renal insufficiency remain particularly challenging to detect in resource-limited settings. Unlike conditions monitored with simple point-of-care tools, diagnosing renal insufficiency requires serum creatinine assays, which depend on laboratory infrastructure and consistent reagent supplies that are frequently lacking. Therefore, identifying factors associated with its incidence is crucial for developing proactive screening strategies. To address these gaps, this study had two primary objectives. First, we determined and compared NCD prevalence at two key time points to characterize the overall disease burden. Second, we aimed to understand the evolving burden of new disease by identifying baseline risk factors for incident NCDs that develop *after* ART initiation, as this represents a distinct clinical challenge. For this incidence analysis, our primary goal was to identify predictors for the onset of any new NCD. Additionally, given the complex, multifactorial etiology of renal decline in this population, we conducted a targeted secondary analysis of factors associated with incident renal insufficiency, for which we hypothesized that receipt of a tenofovir disoproxil fumarate (TDF)-containing ART regimen would be an independent risk factor.

## Methods

### Study setting and population

This is a retrospective cohort study of PLHIV aged 40 years and older who attended routine HIV care visits at the HIV clinics of Hôpital Universitaire Justinien, a public hospital located in Cap Haitien, and Hôpital Sacré-Cœur de Milot, a private nonprofit hospital situated in the town of Milot. Both hospitals are considered high-volume healthcare facilities for the North department and utilize the iSanté electronic medical records (EMR) for patient management. In this EMR, clinical data are entered directly at the point-of-care by attending clinicians during routine HIV clinical visits. Each facility is equipped with an on-site laboratory capable of performing all study-required tests. To be eligible for inclusion in the study, PLHIV must have met the following criteria: (1) were 40 years of age and older at ART initiation; (2) attended at least one clinic visit at either hospital between July 1 and September 30, 2015; (3) had been receiving ART for ≥ 12 months by the time of that visit; and (4) had NCD data available at ART initiation and after ≥ 12 months of ART. For individuals who attended multiple routine HIV clinical visits during the three-month period, data from the visit with the most complete NCD documentation were extracted for the follow-up analysis.

### Data collection and definitions

Data were extracted from the facility EMR between June 9 and November 30, 2016. This dataset included demographic and clinical information (age, sex, height, weight, date of ART initiation, ARV regimen, and duration on ART), cardiometabolic data (blood pressure measurements and documented NCDs such as hypertension and diabetes), and laboratory data (serum creatinine, blood glucose, total cholesterol, and hemoglobin levels). Data were fully anonymized at the time of data extraction. During the subsequent analysis and reporting of results, investigators did not have access to information that could directly identify individual participants.

The presence of NCDs at ART initiation and at the routine clinical visit was determined based on documented clinical diagnoses and/or relevant laboratory values. The primary outcome of this study was defined as having at least one of the following NCDs: hypertension, overweight/obesity, diabetes, dyslipidemia, or renal insufficiency, based on the diagnostic criteria outlined below.

**Hypertension**: Hypertension was defined as a systolic blood pressure of 140 mm Hg or higher, or a diastolic blood pressure of 90 mm Hg or higher on at least two medical visits, in accordance with the Eighth Joint National Committee (JNC 8) criteria, or having a documented prescription of antihypertensive medication [14].

**Overweight and obesity**: The Body Mass Index (BMI) was calculated from the patient’s weight in kilograms divided by the square of the height in meters. Height was measured without shoes, and weight was measured on a weight scale with patients fully dressed but without shoes. Weight was categorized using standardized definitions by the National Institutes of Health: BMI < 18.5 kg/m^2^ as underweight; 18.5- 24.9 kg/m^2^ as normal weight; 25.0-29.9 kg/m^2^ as overweight; and BMI ≥ 30 kg/m^2^ as obese [15].

**Diabetes**: A diagnosis of diabetes was established based on a fasting blood glucose of 126 mg/dL or higher, or the documented prescription of an antidiabetic medication [16].

**Dyslipidemia:** A patient was classified as having dyslipidemia if the total cholesterol level was 240 mg/dL or higher, the low-density lipoprotein (LDL) level was 160 mg/dL or higher, the triglyceride level was 200 mg/dL or higher, or if there was documentation of the patient receiving a lipid-lowering agent [17].

**Renal insufficiency**: The Glomerular Filtration Rate (GFR) was calculated using the Cockcroft-Gault equation [18]. An estimated GFR (eGFR) of less than 60 mL/min/1.73 m^2^ was classified as renal insufficiency [19].

### NCD classification and persistence

NCDs were categorized based on expected clinical persistence and their reliance on ongoing management. Persistent NCDs (hypertension, diabetes, and dyslipidemia) were treated as non-reversible. For these conditions, if a diagnosis was established at ART initiation, the condition was automatically carried forward and counted as present at the follow-up assessment (≥ 12 months on ART). A new NCD diagnosis made at the ≥ 12-month follow-up among those with no documented NCD at ART initiation was classified as an incident case.

Potentially reversible NCDs (renal insufficiency and overweight/obesity) were assessed independently at each time point. For these conditions, NCD status at ART initiation did not carry forward. If an individual met the criteria at ART initiation but their measurements returned to normal thresholds by the follow-up visit, the condition was operationalized as “resolved,” and the individual was not counted as having the condition at follow-up. Conversely, individuals who only met the criteria at the follow-up visit were classified as incident cases.

### Statistical analysis

All statistical analyses were performed using Stata statistical software (version 19.5, StataCorp LLC, College Station, TX). Descriptive statistics were used to summarize the demographic and clinical characteristics of the study population. Continuous variables were reported as means and standard deviations (SD) or medians and interquartile ranges (IQR), while categorical variables were presented as frequencies and percentages.

### Estimation of NCD prevalence and cumulative incidence

The crude prevalence of NCDs at ART initiation and after the ≥ 12 months follow-up visit was calculated as the number of individuals meeting the diagnostic criteria for a specific NCD divided by the total number of individuals. The final analytical cohort of 318 participants had complete data for all demographic and clinical variables at both time points. The only exception was baseline lipid panel data, which were unavailable for 139 (43.7%) individuals at ART initiation. Therefore, all analyses pertaining to dyslipidemia prevalence and incidence were conducted exclusively on the subset of participants with documented lipid measurements. No data imputation methods were utilized. All prevalence estimates were reported alongside their 95% confidence intervals (CIs).

To compare the prevalence of NCDs at these two time points within the same cohort, appropriate paired statistical tests were utilized. For binary categorical NCDs, McNemar’s test was used; for multi-category nominal NCDs, the Stuart-Maxwell test was used. For continuous clinical measurements, the Paired t-test was employed for normally distributed variables, while the Wilcoxon matched-pairs signed-rank test was used for non-normally distributed variables. All statistical tests were two-sided, with a p-value < 0.05 considered statistically significant.

The cumulative incidence of NCDs was determined using an analytical sample restricted to individuals who were free of all defined NCDs at ART initiation. The cumulative incidence for a specific NCD was calculated as the total number of new cases of that NCD identified at the follow-up visit divided by the total number of individuals in the NCD-free analytical sample. As this incidence analysis excludes individuals who already had an NCD at ART initiation, these incident NCD counts should not be used to reconcile the overall study population’s NCD prevalence rates.

### Multivariable logistic regression modeling of incident NCDs

Multivariable logistic regression was used to estimate the odds of developing a new NCD during the follow-up period. To accurately model incidence, the analytical sample for the model was restricted to the “at-risk” population – individuals who were free of all defined NCDs (hypertension, diabetes, hypercholesterolemia, overweight/obesity, and renal insufficiency) at ART initiation. The outcome evaluated was the presence of a new NCD diagnosis at the ≥ 12-month follow-up visit, with predictor variables (e.g., BMI, blood pressure) modeled based on their baseline measurements.

The final multivariable logistic regression model was constructed using a hybrid variable selection approach. Core demographic characteristics (age and sex) and key clinical variables (baseline CD4, ART regimen) were forced into the models *a priori* based on established clinical relevance from prior literature. Additional potential predictors—including duration on ART and baseline hemoglobin—were evaluated in unadjusted bivariate models; those demonstrating an association at a significance threshold of p < 0.20 were retained. Non-significant or highly correlated variables, such as hemoglobin, were excluded from the final multivariable model to ensure parsimony and clinical relevance.

In a secondary analysis, we explored factors associated with the odds of developing incident renal insufficiency using an identically specified logistic regression model. To minimize confounding from pre-existing comorbidities known to impact renal function (e.g., hypertension), the analytical sample for this specific model was restricted to the 109 individuals free of all NCDs at baseline. Incident cases were defined as new diagnoses of renal insufficiency at the ≥ 12-month follow-up within this cohort. The final parsimonious model was adjusted for age group, sex, baseline CD4 count, baseline systolic blood pressure, and ART regimen.

### Ethical considerations

The study was reviewed and approved by the Haiti National Bioethics Committee (Reference number: 1415–3). Because this was a retrospective study utilizing existing electronic medical records, the requirement for informed consent was formally waived by the ethics committee. To ensure patient confidentiality, all data were fully anonymized at the time of extraction, and investigators had no access to personally identifiable information. This activity was reviewed by the U.S. Centers for Disease Control and Prevention (CDC), determined to be non-research, and conducted in accordance with applicable federal law and US CDC policy (See, e.g., 45 C.F.R. part 46, 21 C.F.R. part 56; 42 U.S.C. §241(d); 5 U.S.C. §552a; 44 U.S.C. §3501 et seq.).

## Results

### Demographic and clinical characteristics by NCD status at ART initiation

A total of 3,405 PLHIV were receiving ART at the two health facilities during the study period, among whom 1,956 were 40 years of age or older at the time of ART initiation. Of these, 725 (37.1%) attended a routine HIV clinical visit between July 1 and September 30, 2015. The final analytic cohort comprised 318 individuals who had been on ART for ≥12 months and had complete laboratory data available for both baseline and follow-up time points (Fig 1). Table 1 details the baseline characteristics of the 318 participants, stratified by NCD status at ART initiation (n = 209, 65.7%). Compared to those without NCDs, participants with one or more NCDs had a higher proportion of females (58.9% vs. 44.0%; p = 0.012). Age distribution was similar between the two groups. The NCD group had a significantly higher median CD4 T-cell count (277 vs. 219 cells/mm³; p < 0.001) and a greater proportion of individuals with CD4 counts >350 cells/mm³, and higher median hemoglobin levels (11.9 vs. 11.3 g/dL; p = 0.005). ART regimen and median duration on ART did not differ between the groups. As expected, the clinical measurements of blood pressure, body mass index, eGFR, and blood glucose for the NCD group were consistent with their diagnostic profiles; the complete breakdown of these baseline biological parameters is detailed in Table 1.

**Table 1:** Demographic and clinical characteristics of the 318 PLHIV in the study population by non-communicable disease (NCD) status at ART initiation, North Department, Haiti.

Characteristic	Total N = 318	No NCD N = 109	One or more NCDs N = 209	p-value
**Demographic & Clinical Characteristics**				
Sex, n (%)				
Female	171 (53.8)	48 (44.0)	123 (58.9)	0.012
Male	147 (46.2)	61 (56.0)	86 (41.1)	
Age, median (IQR), years	53 (49-57)	52 (49-57)	53 (49-57)	0.670
Age group, years, n (%)				
40-54	194 (61.0)	69 (63.3)	125 (59.8)	0.540
≥ 55	124 (39.0)	40 (36.7)	84 (40.2)	
CD4 T-cell count, median (IQR), cells/mm^3^	256 (153-377)	219 (117-292)	277 (169-456)	<0.001
CD4 T-cell range, n (%), cells/mm^3^				
< 200	120 (37.7)	53 (48.6)	67 (32.1)	0.003
200-350	110 (34.6)	37 (33.9)	73 (34.9)	
> 350	88 (27.7)	19 (17.4)	69 (33.0)	
Hemoglobin, median (IQR), g/dL	11.6 (10.6-12.6)	11.3 (10.3-12.0)	11.9 (10.7-12.8)	0.005
Antiretroviral regimen				
ZDV-3TC-EFV	174 (54.7)	68 (62.4)	106 (50.7)	0.160
TDF-3TC-EFV	60 (18.9)	14 (12.8)	46 (22.0)	
ZDV-3TC-NVP	51 (16.0)	16 (14.7)	35 (16.7)	
PI-based/Other	33 (10.4)	11 (10.1)	22 (10.5)	
Duration on ART, median (IQR), years	5.5 (3.5-7.3)	5.5 (3.7-7.3)	5.4 (3.5-7.3)	0.870
**NCD-Defining Biological Parameters** ^*^				
Body mass index, median (IQR), kg/m^2^	20.9 (18.6-23.3)	19.7 (18.2-21.8)	21.7 (19.2-24.5)	—
Systolic blood pressure, mean (SD), mm Hg	119 (22)	106 (13)	126 (23)	—
Diastolic blood pressure, mean (SD), mm Hg	76 (13)	69 (9)	79 (14)	—
Glomerular filtration rate, median (IQR), mL/min/1.73 m²	71.0 (56.5-92.7)	80.8 (67.8-108.1)	62.4 (49.6-86.7)	—
Blood glucose, median (IQR), mg/dL	84 (75-96)	83 (70-93)	85 (76.5-97)	—
Total cholesterol, mean (SD), mg/dL	148 (48)	139 (39)	152 (50)	—
LDL cholesterol, mean (SD), mg/dL	91 (53)	85 (48)	93 (55)	—

Abbreviations: 3TC, lamivudine; EFV, efavirenz; IQR, interquartile range; LDL, low-density lipoprotein; NVP, nevirapine; PI, protease inhibitor; SD, standard deviation; TDF, tenofovir disoproxil fumarate; ZDV, zidovudine.^*^P-values are not reported for these parameters, as they are inherently coupled with the diagnostic definitions of the NCD outcomes.

**Fig 1:**
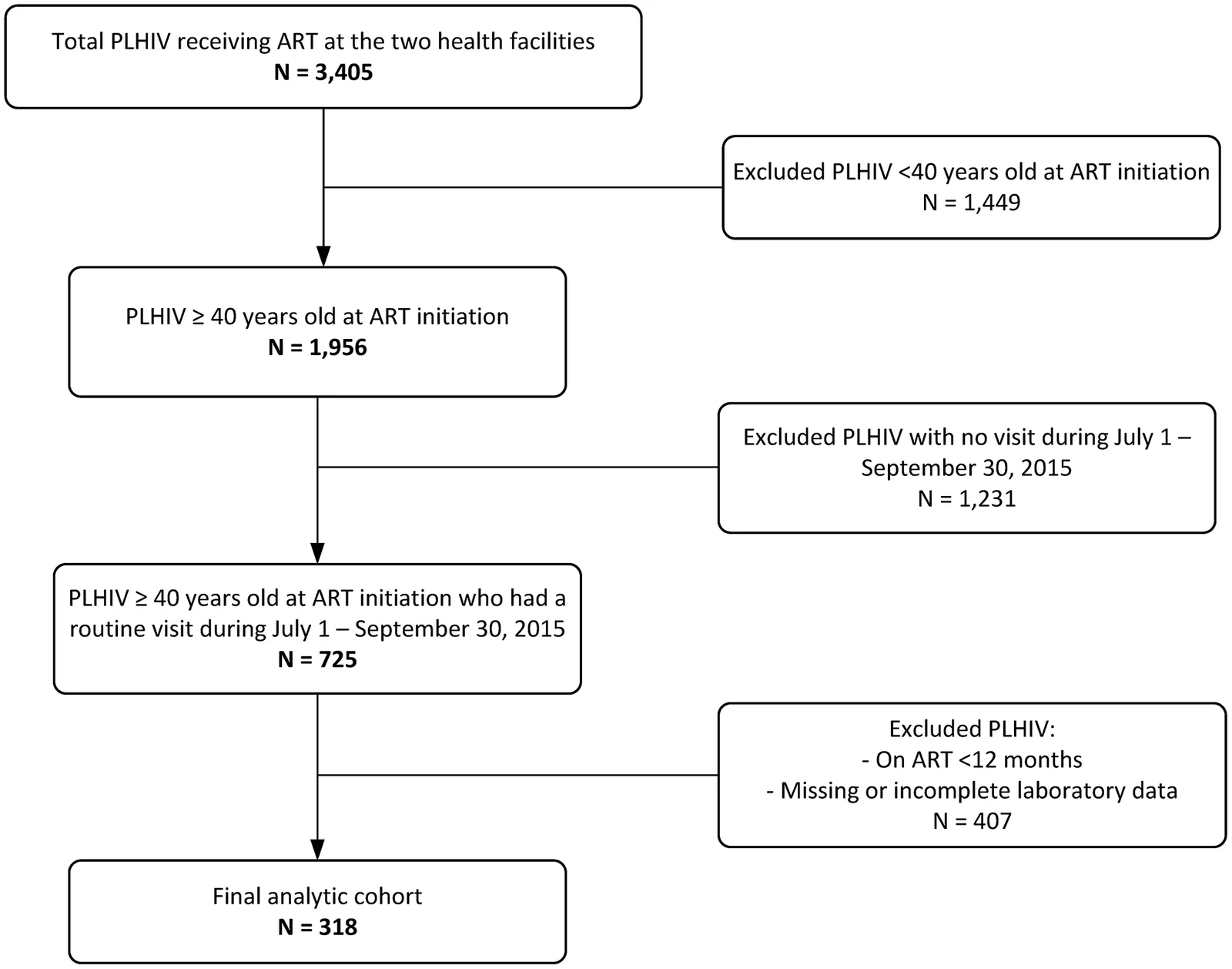
Flow diagram detailing the selection of the 318 PLHIV in the study, North Department, Haiti.

### Demographic and clinical characteristics at ART initiation and after ≥ 12 months of ART

Table 2 shows the demographic and clinical characteristics, as well as the prevalence of specific NCDs, for the same cohort at ART initiation and at the follow-up visit (≥ 12 months on ART). The median duration on ART for the 318 PLHIV was 5.5 years (IQR: 3.5-7.3). Over the follow-up period, the median age increased from 53 years to 58 years, and the proportion of individuals in the 55 years and older age group increased from 39.0% to 74.5%. The median CD4 T-cell count increased from 256 cells/ml at ART initiation to 526 cells/ml at follow-up (p < 0.001), with the proportion of patients with CD4 T-cell count > 350 increasing from 27.7% to 75.8%. Regarding clinical NCD measurements, the median BMI was stable 20.9 kg/m² to 21.4 kg/m². Mean systolic blood pressure rose from 119 mm Hg to 122 mm Hg (p = 0.004), while mean diastolic blood pressure remained unchanged at 76 mm Hg. The median glomerular filtration rate remained stable across the two time points. Median hemoglobin levels increased from 11.6 g/dL to 12.1 g/dL (p < 0.001), and median fasting blood glucose increased from 84 mg/dL to 93 mg/dL (p < 0.001). Mean total cholesterol increased from 148 mg/dL to 178 mg/dL (p < 0.001), while mean LDL cholesterol increased from 91 mg/dL to 104 mg/dL but was not statistically significant (p = 0.099).

**Table 2:** Comparison of patient characteristics and NCD prevalence within the same study cohort at ART initiation and after ≥ 12 months of ART (N = 318), North Department, Haiti.

Characteristic	At ART initiation N = 318	After ≥ 12 months of ART N = 318	p-value^*^
**Demographic and clinical characteristic**			
Age, median (IQR), years	53 (49-57)	58 (54-62)	–
Age group, n (%)			
40–54 years	194 (61.0)	81 (25.5)	–
≥ 55 years	124 (39.0)	237 (74.5)	
CD4 T-cell count, median (IQR), cells/mm^3^	256 (153-377)	521 (358-698)	<0.001
CD4 T-cell range, n (%)			
< 200	120 (37.7)	29 (9.1)	<0.001
200-350	110 (34.6)	48 (15.1)	
> 350	88 (27.7)	241 (75.8)	
Hemoglobin, median (IQR), g/dL	11.6 (10.6-12.6)	12.1 (11.2-13.0)	<0.001
**NCD-Defining biological parameters**			
Body mass index, median (IQR), kg/m^2^	20.9 (18.6-23.3)	21.4 (19.2-23.8)	0.099
Systolic blood pressure, mean (SD), mm Hg	119 (22)	122 (21)	0.004
Diastolic blood pressure, mean (SD), mm Hg	76 (13)	76 (14)	0.950
Glomerular filtration rate, median (IQR), mL/min/1.73 m²	71.0 (56.5-92.7)	72.8 (62.2-87.0)	0.548
Blood glucose, median (IQR), mg/dL	84 (75-96)	93 (85-101)	<0.001
Total cholesterol, mean (SD), mg/dL	148 (48)	178 (50)	<0.001
LDL cholesterol, mean (SD), mg/dL	91 (53)	104 (43)	0.099
**NCD prevalence, % (95% CI)**			
One or more NCDs	65.7% (60.5-70.9)	70.8% (65.8-75.8)	0.103
Number of NCDs			
None	34.3% (29.1-39.5)	29.2% (24.2-34.2)	0.083
Only one NCD	42.5% (37.0-47.9)	42.5% (37.0-47.9)	
Two or more NCDs	23.3% (18.6-27.9)	28.3% (23.4-33.3)	
Overweight/Obese	14.5% (10.6-18.3)	15.7% (11.7-19.7)	0.680
Hypertension	37.1% (31.8-42.4)	47.2% (41.7-52.7)	<0.001
Diabetes mellitus	7.9% (4.9-10.8)	12.6% (9.0-16.3)	<0.001
Renal insufficiency	31.1% (26.0-36.2)	22.6% (18.0-27.2)	0.013
Dyslipidemia^†^	3.4% (0.7-6.0)	11.0% (7.6-14.4)	<0.001

Abbreviations: CI, confidence interval; IQR, interquartile range; LDL, low-density lipoprotein; SD, standard deviation;^*^p-values are not presented for age (continuous or categorical) because change over time was structurally defined by the study’s pre-post design. †N = 179 at ART initiation.

### Prevalence of NCDs among PLHIV at ART initiation and after ≥ 12 months of ART

At ART initiation, 65.7% (n = 209) of the 318 participants had one or more NCDs, which increased to 70.8% (n = 225) after ≥ 12 months of ART (Table 2). At both time points, hypertension was the most prevalent NCD, followed by renal insufficiency and overweight/obesity. The prevalence of persistent NCDs increased significantly during the follow-up period: hypertension rose from 37.1% to 47.2% (p < 0.001), diabetes from 7.9% to 12.6% (p = 0.002), and dyslipidemia from 3.4% (6/179 of those with baseline data) to 11.0% (p < 0.001). For the reversible NCDs, the prevalence of renal insufficiency decreased from 31.1% (n = 99) to 22.6% (n = 72) (p = 0.016), comprising 73 resolved cases, 46 incident cases, and 26 persistent cases. The prevalence of overweight/obesity remained stable, changing from 14.5% (n = 46) to 15.7% (n = 50) (p = 0.658), comprising 45 resolved cases, 49 incident cases, and 1 persistent case.

### Cumulative incidence of NCDs after ≥ 12 months of ART

Among the 109 PLHIV with no documented NCD at ART initiation, 56 (51.4%) developed one or more NCDs after ≥12 months of ART (Table 3). Of these, 41 (37.6%) developed one NCD, while 15 (13.8%) had two or more. The most frequently observed new-onset NCDs were hypertension (21.1%), renal insufficiency (17.4%), and overweight/obesity (15.6%). The cumulative incidences for dyslipidemia and diabetes were 7.3% and 5.5%, respectively.

**Table 3:** Cumulative incidence of specific non-communicable diseases (NCDs) after ≥ 12 months of ART, among 109 PLHIV free of NCDs at ART initiation in North Department, Haiti.

Incident NCD outcome	New cases	Cumulative incidence, % (95% CI)^*^
Number of NCDs		
Only one NCD	41	37.6 (28.5-46.7)
Two or more NCD	15	13.8 (7.3-20.2)
One or more NCDs	56	51.4 (42.0-60.8)
Specific NCDs		
Hypertension	23	21.1 (13.4-28.8)
Renal insufficiency	19	17.4 (10.3-24.6)
Overweight/Obese	17	15.6 (8.8-22.4)
Dyslipidemia	8	7.3 (2.4-12.2)
Diabetes	6	5.5 (1.2-9.8)

Abbreviations: CI, confidence interval; ^*^The denominator for all categories is the 109 individuals who were NCD-free at ART initiation.

### Factors associated with incident NCDs and renal insufficiency

The results of the multivariable logistic regression analyses are presented in Tables 4 and 5. In the primary model for any incident NCD, female sex was the sole independent predictor (AOR: 3.29; 95% CI: 1.40–7.72). In the secondary analysis for incident renal insufficiency, female sex (AOR: 5.88; 95% CI: 1.78–19.50) and elevated baseline systolic blood pressure (AOR: 1.052; 95% CI: 1.005–1.101) were the only significant independent predictors.

**Table 4:** Factors associated with the incidence of one or more non-communicable diseases (NCDs) after ≥ 12 months of ART among 109 PLHIV free of NCDs at ART initiation in North Department, Haiti.

Characteristic	n/N (%)^*^	Unadjusted OR (95% CI)	Adjusted OR (95% CI)
Age group			
40–54 years	17/33 (51.5)	Reference	Reference
≥ 55 years	39/76 (51.3)	0.92 (0.42-2.00)	0.74 (0.31-1.73)
Sex			
Male	25/61 (41.0)	Reference	Reference
Female	31/48 (64.6)	2.63 (1.20-5.74)	3.29 (1.40-7.72)
CD4 T-cell range, cells/mm^3^			
< 200	5/14 (35.7)	Reference	Reference
200-350	9/18 (50.0)	1.02 (0.44-2.35)	1.14 (0.46-2.78)
> 350	42/77 (54.5)	1.07 (0.37-3.06)	1.31 (0.43-3.95)
Antiretroviral regimen			
ZDV-3TC-EFV	34/68 (50.0)	Reference	Reference
TDF-3TC-EFV	9/14 (64.3)	1.80 (0.55-5.93)	1.81 (0.52-6.35)
ZDV-3TC-NVP	7/16 (43.8)	0.78 (0.26-2.33)	0.42 (0.12-1.47)
PI-based/Other	6/11 (54.5)	1.20 (0.33-4.31)	1.22 (0.32-4.64)

Abbreviations: 3TC, lamivudine; CI, confidence interval; EFV, efavirenz; NVP, nevirapine; OR, odds ratio; PI, protease inhibitor; TDF, tenofovir disoproxil fumarate; ZDV, zidovudine; *Column displays the number of incident cases (n) out of the total in that category (N), with the row percentage (%) representing the subgroup incidence.

**Table 5:** Factors associated with the incidence of renal insufficiency after ≥ 12 months of ART among 109 PLHIV free of NCDs at ART initiation in North Department, Haiti.

Characteristic	n/N (%)^*^	Unadjusted OR (95% CI)	Adjusted OR (95% CI)
Age group			
40–54 years	17/33 (51.5)	Reference	Reference
≥ 55 years	39/76 (51.3)	0.58 (0.21-1.59)	2.37 (0.76-7.43)
Sex			
Male	5/61 (8.2)	Reference	Reference
Female	14/48 (29.2)	4.61 (1.53-13.94)	5.88 (1.78-19.50)
Baseline BMI, per 1 kg/m^2^	—	1.30 (1.04-1.61)	—
Baseline SBP, per 1 mm Hg^**^	—	1.035 (0.996-1.075)	1.052 (1.005-1.101)
CD4 T-cell range, cells/mm^3^			
< 200	2/14 (14.3)	Reference	Reference
200-350	2/18 (11.1)	0.83 (0.27-2.53)	0.97 (0.29-3.23)
> 350	15/77 (19.5)	0.81 (0.20-3.31)	0.97 (0.20-4.76)
TDF-containing antiretroviral regimen			
No	14/88 (15.9)	Reference	Reference
Yes	5/21 (23.8)	1.65 (0.52–5.24)	2.94 (0.78-11.10)

Abbreviations: ART, antiretroviral therapy; CI, confidence interval; OR, odds ratio; BMI, body mass index; SBP, systolic blood pressure; TDF, tenofovir disoproxil fumarate; *Column displays the number of incident cases (n) out of the total in that category (N), with the row percentage (%) representing the subgroup incidence. **Estimates for systolic blood pressure are reported to three decimal places to reflect the small incremental unit of measurement (per 1 mmHg) and to provide precise bounds for the statistically significant confidence interval (lower limit > 1.000; p-value = 0.030).

## Discussion

In this retrospective cohort study of older PLHIV in Northern Haiti, we found a high prevalence of NCDs, with over 65% of individuals having one or more NCDs at ART initiation. Notably, the burden of new disease was also substantial: among individuals free of any NCD at baseline, over half developed at least one incident comorbidity within the follow-up period.

During the follow-up period, the prevalence of hypertension and diabetes increased markedly. The high prevalence of hypertension aligns with its significant burden in the older Haitian population [20–22]. Similarly, the diabetes prevalence observed in our cohort (12.6%) closely parallels estimates from the general Haitian population aged 40 years and older, where prevalence rates have been reported to range from 7.2% to 14.4% depending on age group and sex [20,23]. These findings suggest that the metabolic and cardiovascular health burdens among older PLHIV in our study largely run parallel to the broader epidemiological shifts and age-related risk profiles observed in the aging general Haitian population.

Conversely, we observed notable improvements in renal function. The prevalence of renal insufficiency decreased, a net effect driven by the high rate of resolution (73.7%) among those with baseline impairment. This substantial improvement suggests that a significant portion of the baseline renal disease may have been HIV-associated nephropathy, a condition known to respond well to effective ART [24]. In contrast, the overall prevalence of overweight/obesity remained statistically stable, reflecting a balanced rate of both incident and resolved cases. This high rate of resolution for these NCDs suggests that the initiation of ART and integrated clinical management can positively modify the course of these conditions.

Our analysis of risk factors for incident disease yielded a significant and clinically important finding. After adjusting for key demographic and clinical factors, female sex was the sole independent predictor for the onset of any new NCD, increasing the odds by more than threefold. This finding aligns with growing evidence suggesting that women living with HIV may face a disproportionately higher burden of metabolic and cardiovascular complications as they age [25,26]. The underlying drivers for this are likely multifactorial. Biologically, the intersection of HIV-associated chronic inflammation and the menopausal transition may uniquely accelerate metabolic and vascular aging in women [27,28]. Furthermore, socio-structural factors, including differences in health-seeking behaviors, dietary practices, or access to preventative care, may also contribute to this heightened NCD risk [29,30].

This sex-specific risk was further underscored in our secondary analysis of incident renal insufficiency. In that model, female sex remained an independent predictor, increasing the odds of developing renal impairment by nearly sixfold. Furthermore, the renal-specific model confirmed the critical role of managing cardiovascular health, as elevated baseline systolic blood pressure was also significantly associated with future renal decline. Interestingly, although specific ART regimens, including those containing TDF, were associated with large point estimates for excess risk of incident NCDs and incident renal insufficiency (adjusted ORs of 1.81 and 2.94, respectively), these associations did not reach statistical significance in our cohort. This lack of statistical significance likely reflects our small sample size rather than a definitive absence of risk. While our statistically non-significant findings contrast with some global literature highlighting TDF nephrotoxicity [31–33], they align with studies suggesting that the renal impact of TDF may be overshadowed in certain populations when competing risk factors like advancing age and unmanaged hypertension are present [34–36]. Nevertheless, the elevated odds ratios we observed suggest that the potential risk of TDF warrants further investigation in larger samples, even as the clinical urgency of this question may decrease as newer, less nephrotoxic ART regimens are more widely adopted. Ultimately, this underscores the need for integrated cardiovascular management, alongside careful antiretroviral regimen selection, to preserve renal function in aging PLHIV.

### Limitations of the study

This study has several limitations. First, the findings may not be generalizable, as the study was conducted in two hospitals within a single region of Haiti, approximately 10 years ago. Second, our analysis was constrained by the data available in the EMR; we lacked information on important lifestyle risk factors (e.g., diet, physical activity, smoking, and alcohol use), and the reliance on documented diagnoses may have underestimated the true NCD burden. Third, our use of a composite outcome (one or more NCDs), while useful for assessing the overall onset of comorbidity, prevents the identification of disease-specific risk factors. For example, while our model identifies female sex as a strong predictor for developing *any* NCD, it cannot distinguish whether this risk is uniform across all conditions or driven primarily by a single disease, such as hypertension. Finally, our study design was subject to selection bias, as individuals with complete baseline and follow-up data may not generalize to the broader population of older PLHIV who were excluded due to missing clinical measures or loss to follow-up. Consequently, our study had limited temporal resolution, and the reported odds ratios should be interpreted as measures of association, not direct estimates of relative risk.

## Conclusion

This study identified a high prevalence of NCDs among older PLHIV receiving ART in northern Haiti. Our findings emphasize the need for healthcare providers to prioritize integrated care models that combine HIV and NCD management, routine screening, and patient education to improve long-term health outcomes. Additionally, the strong associations of female sex and elevated baseline systolic blood pressure with incident outcomes underscore the clinical value of targeted, routine screenings for high-risk demographics within integrated HIV clinics. Given the evolution of first-line therapy to dolutegravir (DTG)-based regimens—which have their own distinct metabolic considerations—future prospective research is urgently needed to characterize the contemporary burden and drivers of NCDs in the modern status of ART. Such studies are essential to continuously adapt clinical guidelines and ensure the long-term health of aging PLHIV in Haiti and similar settings.

## Supporting information

S1 Data Data file. (XLSX)

S1 Checklist Inclusivity in global research. (DOCX)
